# Personalized Nutrition Approach in Food Allergy: Is It Prime Time Yet?

**DOI:** 10.3390/nu11020359

**Published:** 2019-02-09

**Authors:** Enza D’Auria, Mariette Abrahams, Gian Vincenzo Zuccotti, Carina Venter

**Affiliations:** 1 Department of Pediatrics, Children’s Hospital V. Buzzi, University of Milan, Milan 20154, Italy; gianvincenzo.zuccotti@unimi.it; 2 Faculty of Social Sciences, University of Bradford, Bradford BD7 1DP, UK; mariette@marietteabrahams.com; 3 Section of Allergy and Immunology, Children’s Hospital Colorado, University of Colorado, Aurora, CO 80045, USA; carina.venter@childrenscolorado.org

**Keywords:** food allergy, avoidance diet, nutrition, personalized nutrition, phenotype, microbiome

## Abstract

The prevalence of food allergy appears to be steadily increasing in infants and young children. One of the major challenges of modern clinical nutrition is the implementation of individualized nutritional recommendations. The management of food allergy (FA) has seen major changes in recent years. While strict allergen avoidance is still the key treatment principle, it is increasingly clear that the avoidance diet should be tailored according to the patient FA phenotype. Furthermore, new insights into the gut microbiome and immune system explain the rising interest in tolerance induction and immunomodulation by microbiota-targeted dietary intervention. This review article focuses on the nutritional management of IgE mediated food allergy, mainly focusing on different aspects of the avoidance diet. A personalized approach to managing the food allergic individual is becoming more feasible as we are learning more about diagnostic modalities and allergic phenotypes. However, some unmet needs should be addressed to fully attain this goal.

## 1. Introduction

The true prevalence of food allergy is still unclear: a systematic review of challenge proven food allergy (FA) prevalence in Europe estimates a very low prevalence of FA of 1% [1] compared to single center studies reporting challenge proven prevalence figures of up to 10%. The latest paper on the prevalence of food allergies in children in the USA reports the number of reported FA of 7.6% in children [2] and 10.8% in adults [3].

A small number of foods, such as milk, egg, peanut, tree nuts, wheat, soy, fish, and shellfish, are responsible of most of IgE mediated allergic reactions [4,5]. These reactions are induced by allergenic proteins in the foods and are characterized by rapid onset (usually <2 h). These foods can provoke severe reactions, especially tree nut and peanuts [5,6]. Clinical reactivity to carbohydrates in mammalian meat is an exception—symptoms can be delayed for as long as 6 h [7].

The cornerstone of the management of FA still relies on avoiding the culprit food, since accidental ingestion of the offending food may lead to symptoms including serious and potentially life-threatening reactions, like anaphylaxis [8].

The management of food allergies has seen major transformations in the last decade. It is increasingly clear that the avoidance diet should be tailored according to the patient FA phenotype [9]. Better characterization of FA phenotypes could help to personalize the dietary management of FA by the degree of avoidance required.

Furthermore, there is a greater focus seen on tolerance induction and immunomodulation by microbiota-targeted dietary intervention to allow for greater control of allergies. In the era of precision medicine, the field of precision nutrition involves tailored nutritional recommendations to the individual. To plan personalized nutrition advice for patients with a food allergy, many factors including clinical history, type of allergen, sensitization profiles, threshold level, dietary habits, food preferences, physical activity, microbiome and genotype should all be considered.

In the field of food allergy, some of these factors are better-defined thanks to new diagnostic molecular technologies [10]. Allergen-component resolved diagnostics (CRD) allows differentiating between a true food allergy from pollen-food syndrome or clinically irrelevant sensitization. CRD may predict the risk or severity of allergic reactions to specific food by identifying IgE to epitopes within an allergen source. However, many other components necessary for dietary guidance are poorly understood and need further investigation to be incorporated into clinical practice.

In this review, we will focus on the nutritional management of IgE mediated food allergy, the avoidance diet, state of the art tools/therapies, and the remaining knowledge gap.

## 2. Making an Accurate Diagnosis: The First Step Required to Develop an Avoidance Diet

The first step in the diagnosis of a FA is to distinguish IgE-mediated from non–IgE-mediated reactions. Most IgE caused reactions occur rapidly (minutes up to 2 h after ingestion) with the rare exception [11]. Anaphylaxis is the most serious allergic reaction; it is rapid in onset, life-threatening, and potentially fatal [12]. Different geographical locations show some differences in food allergen triggers for anaphylaxis. A recent one from Spain suggested milk and eggs allergies are more severe than nuts in their population [13].

Unlike IgE mediated, non IgE-mediated reactions are typically delayed from hours to weeks after ingestion of the culprit food(s) [11].

A thourough clinical history is central in diagnosing FA. Components of this history should ideally include food recalls, as well as timing, characteristics, and severity of symptoms. If the history suggests an IgE mediated food allergy, skin prick tests (SPT) or food-specific IgE blood tests can be used to confirm allergy diagnosis [5,14]. A positive test result does not confirm an IgE-mediated allergic reaction, whereas a negative test, with rare exception, eliminates it [15].

In addition to the SPT and specific IgE tests, oral food challenges (OFC) and CRD are important tools for allergy diagnosis. OFC remains the gold standard to confirm clinical reactivity, in most cases [16,17]. Component-resolved diagnostics helps further define specific allergens and reduces misdiagnosis due to cross-reactivity [18,19]. The usefulness of these tools can be explained through the classic example—wheat allergy. Wheat allergy is often over diagnosed, due to the low specificity of wheat IgE testing [20,21]. A patient with a grass pollen allergy may have elevated “wheat IgE levels” while being wheat tolerant [22]. Therefore, both CRD and OFCs should be implemented in children with an SPT or IgE positive wheat allergy. CRD increases the accuracy of wheat allergy diagnosis by identifying the presence of specific IgE to omega-5 gliadin, the antibody highly specific to wheat allergy [23]. Currently, oral provocation with wheat is the reference test for the diagnosis of wheat/cereal allergy as it definitely shows if a child will tolerate wheat.

Additionally, profiling the specific IgE repertoire by CRD may help identify falsely diagnosed allergies in highly polysensitized patients. This can be explained with the case of patients with allergen extract positive but negative genuine components. In children with multiple sensitization to tree nuts, including hazelnut, positive IgE extract but negative IgE genuine component are markers of a probable cross-sensitization with grass pollen. These patients are very likely to be tolerant to hazelnut in vivo [24]. CRD has become a useful tool for diagnosing FA, though the use of these tests varies from country to country.; This technique has some limitations that should be considered. For instance, the allergens are in a recombinant form and not always show the same IgE reactivity that natural allergens. This is even more relevant in food allergy testing as the allergens used in the reagents are processed. Indeed, the oral food challenge (OFC) is the only effective method to confirm the FA diagnosis, although the other preliminary diagnostic techniques could support the diagnosis.

## 3. Risk Assessment and Individual Threshold Level

In general, for IgE mediated-food allergy it is very important to identify patients who are likely to have severe reactions from patients with mild to moderate ones. Unfortunately, as allergy severity is multifactorial, this is difficult. Possible contributors to severe reactions are allergen bioavailability, patient habits (e.g., Exercise [25]), and history of anaphylaxis—although many people who have a history of only mild symptoms can develop anaphylaxis. Allergen-specific IgE levels and CRD may assist in risk assessment as sensitization to some allergenic molecules is more likely to be related to systemic rather than local reactions.

For instance, high levels of casein IgE has been shown to correlate with severe reactions, due to accidental exposure, in cow’s milk allergic children [26]. Similarly, an association between specific IgE to omega-5 gliadin component and severity of reactions during wheat challenge has been reported [21,27]. In peanut allergic children, Eller and Bindslev–Jensen documented that symptom severity elicited during challenge correlated significantly with the levels of Ara h 2 (r(s) ¼ 0.60, P < 0.0001) [28]. However, patients with very low or undetectable sIgE may still experience severe allergic reactions [25,29].

The OFC allows us to ascertain information about individual threshold level can guide the necessary level of food avoidance.

For instance, the challenge food for baked milk contains 1.3 g CM protein (equivalent to 40 mL CM), and children who react during their CM OFC should avoid it completely due to their severe phenotype [30].

Lieberman et al. showed that 66% of the patients with egg allergy undergoing baked egg OFC tolerated baked egg and that most of the reactions were mild and treated with antihistamine alone, regardless of sIgE and/or SPT. [31].

In our opinion, performing OFC with baked milk or egg in a controlled-setting has the potential to greatly improve children’s quality of life [32].

## 4. Avoidance Diet: Towards Personalized Nutrition Advice

Managing food allergies and avoiding food allergic reactions involves an individualized approach to food allergen avoidance while providing sufficient nutrition [33].

An avoidance diet is a complex undertaking that requires education about label reading, cooking, preventing cross-contamination, and communicating information to family, caregivers, friends, and restaurant personnel [34,35]. See Table 1

The standard information that should be provided to all patients includes advice on food labels and relevant labeling laws, hidden allergens, and suitable replacement foods [36]. However, avoidance advice should be individualized considering individual tolerances, cross-reactivity, and specific allergens that drive the reaction. Allergies to novel allergens such as alpha-gal will also require individualized avoidance advice.

### Individualized Allergen Avoidance

#### 4.1.1. Milk and Egg

It is known that a large proportion of children with cow’s milk and egg allergies will be tolerant to baked milk and egg irrespective of the age or population studied [37]. Baked milk or egg-containing foods typically refer to muffins, but other forms such as cookies, waffles, and pancakes have also been suggested. Baked cheese (pizza) has also been suggested for baked milk challenges [38,39,40,41,42,43]. No established guidelines to determine when to challenge have been established, so testing depends on combination of history, sIgE, and skin test results. There is limited consensus about the exact time and temperature of baking/cooking that is required, the need for a wheat/starch matrix, and where the challenge/food reintroduction should be conducted, e.g., hospital/in-office vs. at home [44,45,46]. It is, however, important to realize that some children who react to baked milk or baked egg may experience severe symptoms, requiring epinephrine. [31,32,46]. Risk factors for severe reactions to baked foods need further clarification but may include asthma requiring preventative treatment, multiple IgE mediated food allergies, and a history of anaphylaxis. [45,47]. Baked milk and egg-containing foods are successfully introduced at home in most children’s diets post a negative challenge with good compliance; positively affecting the child’s food and texture repertoire [48]. However, as it is unclear if continued and regular consumption of baked milk and egg-containing foods will speed up tolerance to uncooked milk or egg [49,50], families should not be pressured about frequent intake unnecessarily.

#### 4.1.2. Peanut, Tree Nuts, Seeds

Previously, patients with peanut or tree nut allergies were advised to avoid all nuts, due to the risk of cross-reactivity or possible cross-contact/contamination. However, recent studies indicate that clinical cross-reactivity may be as low as 30% [51]. For instance, walnuts and pecans are highly cross-reactive with each other, but not with peanuts, hazelnuts or almonds Sensitization or clinical allergy may develop after a period of unnecessarily exclusion [52]. The British Society for Allergy and Clinical Immunology (BSACI) guidelines were the first food allergy management guidelines to recommend active inclusion of tolerated nuts in diets of individuals with peanut or tree nut allergy [53,54]. Peanuts are legumes, but allergy to other legumes is generally uncommon among those with peanut allergy, though this does depend on geography and local diet [55,56]. Lupine, pea, and soybean show some apparent cross-reactivity for patients who are highly allergic to peanut, although it is very difficult to separate cross-reactivity from de novo sensitization. The risk of cross-reaction may be higher for lupin than for other beans, particularly in Europe [57,58,59]. In the case of lupine allergy, patients need to be informed about foods containing lupin which may include pies, certain breads, and pastries.

Seeds are being used more often in commercial and gourmet foods—most commonly flaxseed, sesame, sunflower, poppy, pumpkin, and mustard seeds [60]. Sesame and mustard seeds are among the 14 most prevalent allergens in the EU, but not in the US [61]. In Europe, prevalence data indicates sesame and mustard seed allergies are geographically disproportionate: high in some areas (France and Spain), much lower in others (Germany and the Nordic countries) and unknown in Eastern Europe [62]. Mustard and sesame seeds are often hidden in commercial foods, making scrutiny of labels required at all times. Sesame seed allergy is not commonly seen outside of Israel and Europe [63]. In addition to scrutiny of labels, children with sesame allergy should always avoid sesame oil as it is cold/expeller pressed [64].

#### 4.1.3. Fruit and Vegetable Allergies

Allergies to fruit and vegetables, in particular, require individualized advice as symptoms range from milder symptoms triggered by pollen-food syndrome (PFS, secondary IgE mediated food allergy) to more severe symptoms triggered by lipid transfer protein syndrome (LTP, primary IgE mediated food allergy) [65]. It is important to differentiate between these two presentations of fruit and vegetable allergies as that will direct the dietary advice given. With PFS, cooked, canned, baked, microwaved fruit and vegetables are allowed, whereas fruit/vegetable should be completely avoided in the case of LTP allergies. The degree to which cross-reactive fruit and vegetables (including soy and nuts) should be avoided requires careful diagnostic evaluation as blanket avoidance advice is not advocated [66,67,68].

#### 4.1.4. Fish and Shellfish Allergy

It is important to distinguish between fish and shellfish (crustacean and mollusks) allergies. Fish and shellfish allergies may co-exist [69] but the main allergens differ, and cross-reactivity between fish and shellfish is unlikely. The main allergen in fish is β parvalbumin; in the case of shellfish, the major allergen is tropomyosin [70]. Additionally, allergy to a certain fish or shellfish does not imply allergies to all species in that particular group [71,72]. Subjects who suffer from fish allergy have only about a 50% probability of being cross-reactive to another fish species. This is significantly lower than those with shellfish allergies, who have up to a 75% chance of cross-reactivity [15]. In addition to the allergens derived from fish themselves, fish contaminants, such as the parasite Anisakis, can also cause allergic reactions, meaning Anisakis allergy can be falsely diagnosed as a fish allergy. In particular, Anisakis allergy correlated to prevalence of parasitic infection in fish—for example, in Spain and Southern Italy, there is a higher prevalence of Anisakis allergy due to moderately frequent Anisakis infection. These allergic patients develop IgE against tropomyosin from Anisakis. As always, sensitization depends in part on the consumption pattern of fish (cooked, undercooked or raw) and the infection pattern of fish in the local region [73].

#### 4.1.5. Alpha-Galactosidase

Alpha galactosidase (Alpha-gal) allergy is characterized by delayed (4 to 6 h after the ingestion) hypersensitivity reactions to mammalian meats and is mediated by IgE antibodies to the oligosaccharide galactose-alpha 1,3-galactose. It requires avoidance of mammalian meats and their organ meat. Some individuals also need to avoid ice-cream, milk, and milk products but the degree of avoidance and foods being avoided should be discussed with the allergist. This decision can be made based on past history of reactions or tolerance [74,75]. Where the history is unclear, or the food has not been eaten in the past, an oral food challenge can be conducted [76].

## 5. Nutritional Impact of Food Allergies: Growth and Nutrient Intake

There is rising concern that children with FA have an insufficient nutrient intake or nutrient imbalance leading to adverse health implications. Data published over the past few years indicates that children with food allergies (IgE, non-IgE, and mixed presentations of IgE and non-IgE) show growth impairment, both in weight and length. They are often underweight [77], and in the case of chronic malnutrition, they become stunted, e.g., a child who is too short for his/her age [78,79]. However, excessive weight gain has also been reported in children with food allergies, but poorly researched [77,80,81]. A recent international survey conducted by Meyer et al. [82] included 430 patients from twelve allergy centers world-wide. The pooled data indicated that 6% were underweight, 9% stunted, 5% undernourished, and 3–5% were overweight. In this study, growth impairments varied by allergy profile. Children with cow’s milk allergy (CMA) had a lower weight for age z-score, as a result of acute malnutrition or “wasting”; children with mixed IgE and non-IgE mediated FA were stunted, and children with only non-IgE FA were underweight with lower body mass index (BMI). Very different growth patterns were observed between children from different countries. Atopic comorbidities did not affect growth.

Avoidance diets required for FA management place children at risk for potential inadequate nutrition. In this regard, a number of studies have investigated the nutritional adequacy of elimination diets. However, most of them have been conducted in young children aged six months to four years. Children with food allergies (IgE, non-IgE, and mixed presentations of IgE and non-IgE) are also at higher risk of insufficient intake of protein, calories, vitamins, and minerals [83,84,85,86,87]. The micronutrients implicated are iodine, calcium, and vitamin D, especially in children with CMA [83,88,89]. However, it has been shown that children with cow’s milk allergies or multiple food allergies are able to achieve similar mean intakes of nutrients as healthy children when receiving nutrition counselling and substitution of nutritionally equivalent foods [78,83,90,91,92].

Limited data exist on dietary intake in teenagers and adults with food allergies, with contrasting results [93,94]. One study reports, higher intakes of calcium, iron, folate, and vitamin E have been demonstrated in participants >20 years with food allergy [44]. Conversely, lower intakes of calcium and phosphorous have been reported in young adults with CMA, with one study reporting that 27% were at risk of osteoporosis [48]. Maslin et al. showed no significant difference between these two groups and control groups with the intake of calcium. Iron, copper, zinc, selenium, and iodine were below the Recommended National Intakes (RNI) for both groups and their controls [94]. There are currently no data on BMI status on adults with IgE mediated food allergy. These factors need to be considered when providing nutrition advice to children and adults with food allergies. Although information on healthy eating is important, consideration to vitamin and mineral supplementation in hypoallergenic formulas in the case of children should be given [84,95]. Nutritional counselling and monitoring growth and development are crucial in the management of FA, as the avoidance diet may affect the well-being of FA patients (see Table 2).

## 6. Food Behaviour and Preferences

In children with FA, the development of their food habits and preferences takes place in the context of their chronic condition. Since parents have the main responsibility for the dietary management of their child’s food allergies [96], their parenting style and the way they interact with the child during feedings both have an effect on a child’s food habits [97]. A child’s food allergies add a burden to parents [98]. Food refusal has also been shown to occur in toddlers with food allergies [99] and more specifically eosinophilic gastrointestinal disease [100]. Additionally, a study on children aged 5 to 14 years in France showed that children who have outgrown their food allergies are more reluctant to try new foods than their siblings [101]. Food neophobia and refusal could result from unnecessarily high dietary restrictions that parents place on their children due to increased anxiety and fear of an allergic reaction [102]. The long-term effects of avoidance diet on food behavior and preferences needs further investigation.

Food choice behavioral problems have been documented in older children or adults with food allergies. Teenagers with food allergies, strive to eat the same foods as their peers, often leading to risk taking behavior. However, they reported reluctance to try new foods when away from home. In contrast to the non-food allergic teens, those with food allergies felt that parental control over food intake was to protect them [103].

Adults with FA felt that their allergies limited them from the pleasure of eating and they often found it difficult to find safe foods. They also felt that the need to be constantly organized to have safe foods available was a burden [104].

## 7. Microbiota-Diet and Genetic Factors: A Complex and Still Unknown Interplay

FA is thought to be the result of a disruption of mucosal immunological tolerance, due to dietary factors, gut microbiota, and interactions between them [105]. Different bacterial taxa may be associated with different food allergy subphenotyes. Differences in gut microbiome have been observed in subjects with tree-nut allergy in respect to those with cow’s milk allergy [106,107]. The observed differences may however be influenced by age, population, sex and diet. Furthermore, recent data indicate that for cow’s milk allergy, the microbiome differs between those children who are sensitized vs. not sensitized [108], those with clinical allergy vs. those with no allergy [109], and those who develop tolerance vs. those who do not [110]. Overall, these findings suggest the possibility to manipulate the gut microbiota with preventive or therapeutic purposes.

Data in pediatric studies indicate that certain pre and probiotics tested may address dysbiosis [111] and may even induce tolerance development [112]. More clinical trials regarding the use of pre and probiotics in the management of food allergies are needed before clinical recommendations can be made. These studies should also consider genetic background and age in their design. Another important issue to be considered is that the gut microbiome composition and diversity can be modulated by host genetic profiling [113]. A host’s genetic composition is able to modulate their gut microbiota, which is another paramount area of study [114].

Whether diet diversity may improve dysbiosis and microbial diversity in those with food allergies remains to be seen [115].

Further studies need to investigate the complex interplay between the host genetic components and environmental factors, including the microbiota and diet, in the pathogenesis and expression of food allergy that is still largely unknown.

## 8. The Technology Revolution in FA Management

Increasingly, personalized devices to aid in allergen detection have been invented, and the industry has grown rapidly over the last decade [116]. These technologies have resulted both from increased demand for transparency of product information and scientific advancements. [117]. The rapid drop in the price of personalised nutrition devices has resulted in mass accessibility [118]. Deciphering food labels is a difficult task and for those with allergies, a daily chore that if done incorrectly, can lead to negative and possibly fatal outcomes [119,120].

New digital technologies have started to appear on the market that attempts to address the daily challenges families face when choosing products for a child with allergies. For a full review of technologies involved in portable allergy products, we refer readers to the comprehensive article by Ross, G.M.S [121]. There have been a number of technology services advising about potential risks related to food composition. For concerned consumers, having instant access to information can remove the guesswork and can potentially save time. However, there are no validated, personalized systems for testing individual meals for specific food source products. It is also noteworthy that sometimes component recipes change and accuracy as well of lack of clinical validation of these products are issues frequently raised.

**Figure 1:**
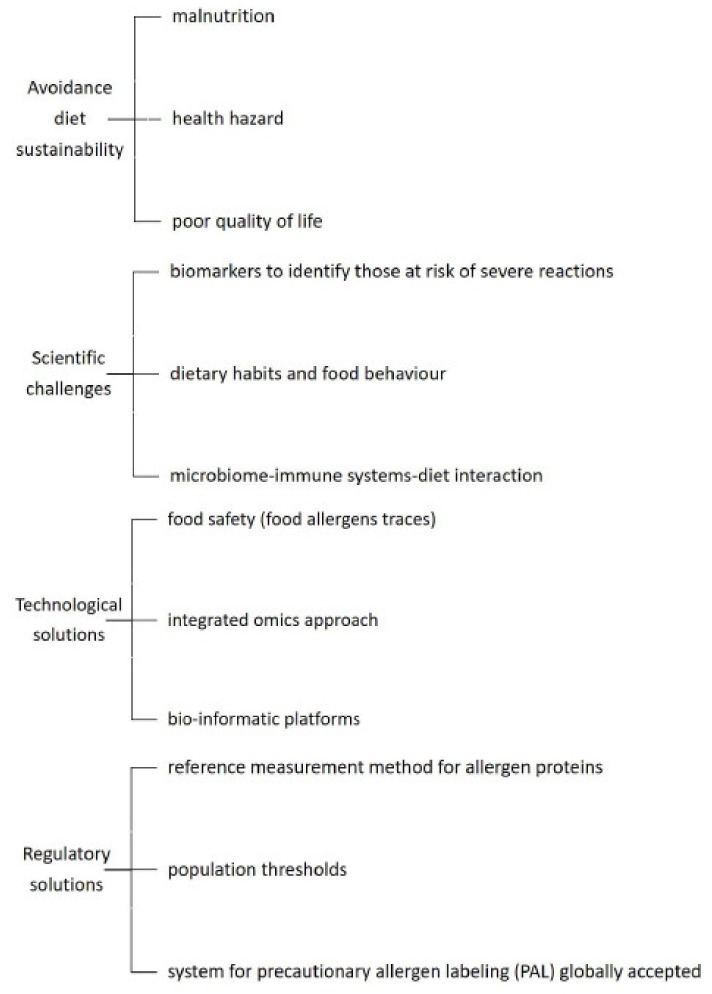
Nutrition approach: unmet needs.

With such rapid advances in the scientific and technology industry, it is, however, important to have comprehensive communication between consumer advocates, the food industry, and the clinicians to help improve avoidance of allergens by technical fixes, while being fully aware of the limitations and current lack of validation of these products in a variety of matrices or in foods with multiple ingredients (see Figure 1). What is clear, is that management of allergies will require the intervention of a specialist multidisciplinary team with registered dietitians playing a key role in supporting families while staying abreast of new technologies [122].

Some examples of products currently available on the market, outlining their pros, cons and future considerations, are listed below (Table 3).

## 9. Conclusions

A personalized approach to managing the food allergic individual is becoming more feasible as we are learning more about diagnostic modalities and allergic phenotypes. The availability of specialized foods and technology are increasing which also enables the clinicians to provide personalized advice. A multidisciplinary team approach, including a dietitian, is crucial to provide individualized recommendations to patients.

## Figures and Tables

**Table 1:** Nutritional management according to risk assessment: What are the challenges?

Challenges of the Nutritional Management According to Risk Assessment
- local availability of food - lack of understanding about foods to be avoided - unexpected allergens in foods - prepacked foods with inadequate allergen labeling - defining “baked” milk and egg - identify the “eliciting dose” - risks of over restrictive diet - potential long-term effects on health and quality of life

**Table 2:** Effect of avoidance diet on patients.

Effect of Avoidance Diet
- poor growth - micronutrient deficiencies - altered taste perception - long term effects on food preferences and choices - reduced quality of life

**Table 3:** Personalized nutrition offering for Food allergies.

Currently Available Resources or Tools	Description	Pros	Cons	Future directions
Apps	Smartwithfood^TM^, Spoonguru^TM^, Foodmaestro^TM^, Whisk^TM^. These apps are available free to consumers. Through barcode scanning, image recognition, natural language processing and machine learning technology, consumers can obtain instant information whether a product contains allergens.	These app scanners provide quick results that are easy to understand and can always be on hand. They can provide peace of mind as a second line. The platforms rely on food manufacturers to provide accurate product information in terms of their recipes.	The app only reports on a limited number of allergens. The app is not a medical device and, therefore, cannot replace a medical professional’s advice; consumers should always ask questions and always check the food label.	Apps should increase the number of allergens they have information about. New products could ideally be developed based on the popularity of scanned products.
Food scanners	Scanners such as Tellspec^TM^, Scioscan^TM^ and Nima^TM^ are handheld, mobile devices that use hyperspectral or imaging technology to analyse nutritional information and detect allergens.	These scanners are small, provide quick results that are easy to understand. They can provide peace of mind as a second line. These products may provide some reassurance once standard allergen avoidance advice has been followed but should NOT be used instead of advice provided by the allergist or dietitian.	Costs can be prohibitive. It is not a medical device and, therefore, consultation with a healthcare professional is still required. Concerns have been raised about the accuracy in detecting allergens (Popping et al., 2017). Scanners work best with homogenous solid products. For example, testing may be highly inaccurate in foods with multiple ingredients or high-fat matrices. It is not clear who holds the data on these products.	These tools need to be clinically validated These tools need to comply with medical devices regulation
Wearable devices	Such as Allergy Amulet^TM^ is a device that is worn as a necklace and works by inserting strips into food, available in 2019.	A mobile and attractive device that provides instant results. These products may provide some reassurance once standard allergen avoidance advice has been followed.	It is not a medical device It is important the consumers read labels and ask about ingredients to the dietitian. Have not been validated for accuracy	Needs to be clinically validated. In the future, potentially sensors or implants could detect from a nanoparticle of food.
CRISPR	Is the new technology which enables DNA of food (and humans) to be edited. This means that new foods and products can be developed where the culprit allergen’s DNA has been edited without the devastating effects.	Consumers with allergies will have a wider variety of foods to eat	Technology is still expensive. Some allergens can be removed. It is not clear how differentiating appropriately altered foods from native food sources. For some allergenic sources, such as wheat, the genetic complexity of the crop is unlikely to allow simple genetic knockout of allergenic genes.	Current lack of understanding of the long-term impact of eating gene-edited foods. Extensive public education will be required.
